# A Rare Cause of Internal Jugular Vein Thrombosis: Deep Tissue Massage

**DOI:** 10.31486/toj.25.0002

**Published:** 2025

**Authors:** Matthew Sanborn, Matthew Koury, Alisha Maity, Zonera Ali

**Affiliations:** ^1^ Department of Internal Medicine, Lankenau Medical Center, Wynnewood, PA; ^2^ Department of Hematology and Oncology, Lankenau Medical Center, Wynnewood, PA

**Keywords:** *Anticoagulants*, *massage*, *neck injuries*, *thrombosis*, *upper extremity deep vein thrombosis*

## Abstract

**Background:**

Upper extremity deep vein thrombosis accounts for a small percentage of all deep vein thrombosis cases, and internal jugular vein thrombosis is a rare subset within that category. Typically, internal jugular vein thrombosis is found in patients with predisposing risk factors such as infections, local trauma secondary to venous catheterization, intravenous drug use, and malignancy.

**Case Report:**

We describe the case of a patient with no typical risk factors for thrombus formation who developed internal jugular vein thrombosis following a deep tissue massage with a focus on the neck area. The patient developed right arm pain shortly after the massage, prompting her to present to the emergency department. Ultrasound revealed a nonocclusive thrombus in the right internal jugular vein. The patient was started on rivaroxaban, and her symptoms resolved.

**Conclusion:**

Deep tissue massage is not a well-established cause of internal jugular vein thrombosis. This case emphasizes the importance of obtaining a thorough patient history to assess for uncommon traumatic causes of internal jugular vein thrombosis.

## INTRODUCTION

Upper extremity deep vein thrombosis accounts for 4.4% of all deep vein thrombosis cases, with internal jugular vein thrombosis representing a rare subset within this category.^1^ Infections, local trauma secondary to venous catheterization, intravenous drug use, and malignancy are well-described risk factors for internal jugular vein thrombosis.^2^ We present a patient who developed painful internal jugular vein thrombosis secondary to deep tissue massage with a focus on the neck area.

## CASE REPORT

A female in her 50s with no significant medical history presented to the emergency department (ED) for worsening pain in her right arm 1 day after a deep tissue massage that involved excessive and painful manipulation of her neck. The pain began in the patient's right shoulder, progressed to the entire right arm, and was accompanied by intermittent paresthesia affecting the second to fourth digits, suggesting venous congestion or secondary inflammation affecting neurovascular structures near the thoracic outlet. The patient sought emergency medical attention because nonsteroidal anti-inflammatory drugs provided inadequate pain management. The patient's body mass index was 24.5 kg/m^2^, she was physically active, and she ate a healthy diet. She had no personal or family history of thrombosis.

On physical examination, musculoskeletal tenderness was evident in the posterior aspect of the right arm. Distal pulses were present bilaterally, and motor and sensory functions were preserved without peripheral nerve deficits. Basic metabolic panel, complete blood count, cardiac troponin, international normalized ratio, and partial thromboplastin time were unremarkable. Ultrasound demonstrated a nonocclusive thrombus in the right internal jugular vein, and the diagnosis was confirmed by computed tomography angiography (CTA). CTA detected no chest mass, extrinsic compression of the internal jugular vein, or evidence of pulmonary embolic disease. The patient was started on rivaroxaban 15 mg twice daily for 21 days, followed by 20 mg once daily. The patient was discharged from the ED with referrals to outpatient Vascular Surgery and Hematology.

The patient was evaluated by Vascular Surgery 1 week later, at which time the pain had resolved. Ultrasound performed in the clinic confirmed that the thrombus was still present, but no surgical intervention was recommended. The patient was evaluated by Hematology 1 week later, and an inherited hypercoagulability workup was performed. Anticardiolipin antibodies, antithrombin III activity, homocysteine, beta-2 glycoprotein 1 antibodies, factor V Leiden mutations, lupus anticoagulant, and prothrombin gene analysis were all unremarkable.

The patient returned for a 3-month follow-up visit with the hematologist. Repeat venous ultrasound of the right arm showed no evidence of residual thrombosis. Because the patient had completed 3 months of rivaroxaban and the inherited thrombophilia workup was unrevealing, anticoagulation was discontinued.

## DISCUSSION

Thrombosis in the head and neck region is uncommon; the majority of cases occur in the lower extremities. Typically, internal jugular vein thrombosis associated with trauma arises from direct injury or catheter-related complications.^2^ Jugular vein thrombosis has been reported in the absence of traditional risk factors, including a case linked to deep tissue massage that caused external jugular vein thrombosis and a case of cervical chiropractic manipulation that led to internal jugular vein thrombosis.^3,4^ Another case described a presentation similar to ours in which a 35-year-old male with no thrombotic risk factors and a negative hypercoagulability workup developed internal jugular vein thrombosis following shiatsu massage.^5^ That patient waited a month after symptom onset to seek medical treatment and presented with neck swelling, while our patient sought medical treatment the day following the massage.

The cause of our patient's internal jugular vein thrombosis following neck massage can be analyzed through the Virchow triad, the factors that lead to an increased risk of thrombosis: blood stasis, endothelial injury, and a hypercoagulable state. Direct trauma to the internal jugular vein, such as from central venous catheters, can provoke local inflammation, reduce blood flow, and increase the risk of thrombus formation because of endothelial damage and cell adhesion.^6^ However, the specific mechanisms by which a neck massage could induce internal jugular vein thrombosis remain speculative. Theories suggest that massage could cause venous stasis directly or through subsequent inflammation, as noted by Pastori et al.^2^ Dislocation of an existing clot is another possibility, but this explanation is unlikely in our patient because of the localized nature of the massage, the absence of identifiable thrombotic risk factors, and her negative hypercoagulability workup. Our patient also had no identifiable acquired risk factors such as obesity, sedentary lifestyle, or use of birth control that would increase her risk of a thrombus. Given that the patient had resolution of her thrombus and no concerning physical examination findings, further investigation into malignancy was not indicated.

This case details the importance of considering less common etiologies such as deep tissue massage in the differential diagnosis of internal jugular vein thrombosis. The case also highlights the need for increased clinical investigation and further research to determine the mechanisms by which manipulative neck therapies might elicit internal jugular vein thrombosis. This understanding is necessary for guiding treatment durations and preventing recurrent thrombotic events. Furthermore, patients should be cautious when having deep tissue massage performed and should likely avoid excessive neck manipulation.

## CONCLUSION

Deep tissue massage and physical manipulation of the neck can cause internal jugular vein thrombosis, highlighting the importance of recognizing uncommon traumatic triggers. Identifying the cause of a deep vein thrombus is important for determining the proper treatment duration. The mechanism for how deep tissue massage can elicit an internal jugular vein thrombus remains unclear and warrants further investigation.

## Notes

*This article meets the Accreditation Council for Graduate Medical Education and the American Board of Medical Specialties Maintenance of Certification competencies for Patient Care and Medical Knowledge.*
